# A benchmark of transposon insertion detection tools using real data

**DOI:** 10.1186/s13100-019-0197-9

**Published:** 2019-12-30

**Authors:** Pol Vendrell-Mir, Fabio Barteri, Miriam Merenciano, Josefa González, Josep M. Casacuberta, Raúl Castanera

**Affiliations:** 1 Centre for Research in Agricultural Genomics CSIC-IRTA-UAB-UB, Campus UAB, Edifici CRAG, Bellaterra, 08193 Barcelona, Spain; 2 Institute of Evolutionary Biology (CSIC-Universitat Pompeu Fabra), Passeig Maritim Barceloneta 37-49, 08003 Barcelona, Spain

**Keywords:** Benchmark, Transposable elements, Polymorphism, Transposon insertion, Resequencing

## Abstract

**Background:**

Transposable elements (TEs) are an important source of genomic variability in eukaryotic genomes. Their activity impacts genome architecture and gene expression and can lead to drastic phenotypic changes. Therefore, identifying TE polymorphisms is key to better understand the link between genotype and phenotype. However, most genotype-to-phenotype analyses have concentrated on single nucleotide polymorphisms as they are easier to reliable detect using short-read data. Many bioinformatic tools have been developed to identify transposon insertions from resequencing data using short reads. Nevertheless, the performance of most of these tools has been tested using simulated insertions, which do not accurately reproduce the complexity of natural insertions.

**Results:**

We have overcome this limitation by building a dataset of insertions from the comparison of two high-quality rice genomes, followed by extensive manual curation. This dataset contains validated insertions of two very different types of TEs, LTR-retrotransposons and MITEs. Using this dataset, we have benchmarked the sensitivity and precision of 12 commonly used tools, and our results suggest that in general their sensitivity was previously overestimated when using simulated data. Our results also show that, increasing coverage leads to a better sensitivity but with a cost in precision. Moreover, we found important differences in tool performance, with some tools performing better on a specific type of TEs. We have also used two sets of experimentally validated insertions in *Drosophila* and humans and show that this trend is maintained in genomes of different size and complexity.

**Conclusions:**

We discuss the possible choice of tools depending on the goals of the study and show that the appropriate combination of tools could be an option for most approaches, increasing the sensitivity while maintaining a good precision.

## Background

Transposable elements (TEs) constitute a very important fraction of eukaryotic genomes, and their ability to transpose, excise and produce complex genomic rearrangements make them a key source of genomic diversity. Previous work done over the last decades has uncovered their enormous potential as gene regulators, a role that TEs play through a variety of genetic and epigenetic mechanisms [12, 43]. Certain TEs, such as Long Terminal repeat (LTR)-retrotransposon carry their own promoters, and their insertion close to genes can generate new gene expression patterns. In addition, TEs, and in particular LTR-retrotransposons and MITEs (Miniature Inverted Transposable Elements), have been shown to contain transcription factor binding sites, which can be mobilized by transposition rewiring new genes into pre-existing transcriptional networks [5, 12, 20]. As a consequence, TEs have the potentiality to generate important genomic and transcriptional variability, and the interest in these elements has drastically increased in the last years.

Due to their repetitive nature and their sequence diversity, the annotation of TEs is more complex than that of protein coding genes. Nevertheless, thanks to the development of tools such as Repeatmasker (http://www.repeatmasker.org) and sophisticated pipelines such as REPET [16], methodologies of TE detection and annotation in assembled genomes are today robust. The availability of high-quality reference genomes coupled with the exponential increment of resequencing data has boosted our capacity to evaluate intraspecific variability. By obtaining accurate maps of genetic variation, characterizing the genetic basis of phenotypic variance is now possible at a genome-wide scale thanks to association studies (GWAS). Until now, most of the efforts have been focused on analyzing the variability at the nucleotide level (SNPs, single nucleotide polymorphisms), as there are robust algorithms to perform variant calling. However, TEs generate an important part of the genetic variability present in a particular species. Moreover, the timing of occurrence of TE and SNP mutations is different, as the former can amplify in bursts generating a great amount of diversity in short periods of time, whereas SNP mutation rates are more constant in time. Therefore, the identification of Transposon Insertion Polymorphisms (TIPs) is of high interest. Nevertheless, our capacity to accurately identify TIPs using re-sequencing data is hampered by the structural complexity of TEs.

In the last few years, many laboratories have developed bioinformatic tools to look for TIPs and have started to analyze their impact in intra-species variability, including crop plants [7, 10, 42]. There are two main approaches that can be used to detect TIPs in whole-genome sequence data: i) inference from discordant read-pair mappings, and ii) clustering of ‘split’ reads sharing common alignment junctions [2, 15]. Most of the recently developed tools incorporate both methodologies, and in some cases TIPs have been experimentally validated [27]. Moreover, in some cases the authors have evaluated their sensitivity and precision (also known as positive predictive value) [11, 24]. However, in most cases these evaluations were performed by generating simulated insertions that are randomly placed in the genome, and then used to compare with tool predictions. Simulated insertions are far from representing the complexity of “natural” TIPs, as many of their features are difficult or impossible to mimic accurately (i.e.: element degeneration, nested insertions, insertion preferences, etc.). As a consequence, the benchmarks done with simulated data tend to overestimate performance of the tools analyzed [21]. An example of such benchmarks is the one reported by the developers of McClintock, a pipeline that integrates six tools [36] (Table 1). In their study, the authors provided a detailed comparison of their component’s performance in sensitivity and positional accuracy based on simulated LTR-retrotransposon insertions, which also includes some real resequencing data, in the yeast *Saccharomyces cerevisiae*. In spite of the interest of such comparative analysis, the direct translation of these results to other eukaryotic models with bigger and more repetitive genomes is uncertain. This is especially relevant as *S. cerevisiae* contains only 51 full LTR-retrotransposons in the whole genome [8], whereas in most plant and animal genomes the LTR-retrotransposon load is several orders of magnitude higher. Also, a recent study focused on simulated but also real human AluY, L1 and SVA families revealed huge differences in the ability of seven tools to detect TIPs [41]. In spite of the importance of these families for human research, they do not represent the diversity of the TE landscape of other animals and plants, which is far more complex, with many families from different orders being potentially active, and where the amount of truncated non-autonomous elements greatly outnumbers the active copies.

**Table 1:** Tools selected for the benchmark of TE insertions

Tool	Target	Prediction	Input	Output format	Perceived difficulty		Manual
					Installation	Input preparation	
RelocaTE2	Non-reference insertions	All families	fastq	gff file	Easy	Easy	https://github.com/JinfengChen/RelocaTE2
Jitterbug	Non-reference insertions	All families	Bam	gff file	Medium	Medium	https://github.com/elzbth/jitterbug
Retroseq ^a^	Non-reference insertions	All families	Bam	vcf file	Easy	Difficult	https://github.com/tk2/RetroSeq/wiki/RetroSeq-Tutorial
ITIS	Non-reference insertions	Single-family	fastq	Bed file	Easy	Medium	https://github.com/Chuan-Jiang/ITIS
MELT	Reference and non-reference insertion	Single-family	Bam	vcf file	Easy	Medium	http://melt.igs.umaryland.edu/manual.php
PopoolationTE2	Reference and non-reference insertions	All families	fastq	Tool-specific	Easy	Easy	https://sourceforge.net/projects/popoolation-te2/
Teflon	Reference and non-reference insertions	All families	fastq	Tool-specific	Medium	Medium	https://github.com/jradrion/TEFLoN
Trackposon	Reference and non-reference insertions	Single-family	fastq	Bed file	Easy	Easy	http://gamay.univ-perp.fr/~Panaudlab/TRACKPOSON.tar.gz
TEMP ^a^	Reference and non-reference insertions	All families	Bam	Tool-specific	Easy	Difficult	https://github.com/JialiUMassWengLab/TEMP/blob/master/Manual
TE-locate ^a^	Reference and non-reference insertions	All families	Sam	Tool-specific	Easy	Difficult	https://sourceforge.net/projects/te-locate/
PopoolationTE ^a^	Reference and non-reference insertions	All families	fastq	Tool-specific	Easy	Difficult	https://sourceforge.net/p/popoolationte/wiki/Workflow/
ngs_te_mapper ^a^	Reference and non-reference insertions	All families	fastq	Bed file	Easy	Difficult	https://github.com/bergmanlab/ngs_te_mapper
McClintock	Reference and non-reference insertion	All families	fastq	Bed file	Easy	Difficult	https://github.com/bergmanlab/mcclintock

^a^ These tools were run as part of the McClintock pipeline. Perceived difficulty refers to McClintock and not the original methods

Installation: Easy = available in Conda, or automatic / semi-automatic installation. Medium = Needs several dependencies or specific versions of packages that need manual installation. Input preparation: Easy = can be run using common formats (ie fasta, bed) without the need of specific formatting. Medium = Needs specific formatting. Difficult = Needs very specific formatting

In plants, TEs are at the origin of important agronomic traits, such as apical dominance in maize [45], the skin and flesh colors in grape [28] and blood oranges [4]. Different efforts have been made recently to identify TIPs that could be responsible for important variability in plants. Carpentier *et. al* [7] screened the presence of 32 rice LTR-retrotransposon families in the 3000-rice genome dataset and uncovered more than 50,000 TIPs, most of them occurring at a very low frequency, which is indicative of recent activity. Besides LTR-retrotransposons, MITEs are probably the most prevalent group of transposons in plants, including rice, where they have experienced recent massive amplification bursts [10, 35]. MITEs are structurally very different from LTR-retrotransposons, as they are non-autonomous, usually non-coding, and relatively small. They are of particular interest because they tend to integrate close to genes and may carry regulatory domains [20], having the potential to create or rewire regulatory networks [12]. In the present study, we have taken advantage of the existence of several high-quality assembled genomes of different rice varieties to create a validated dataset of natural LTR-retrotransposon and MITE insertions obtained by direct comparison between the assembled genomes (Nipponbare and MH63), that we have used to benchmark the performance of 12 TIP calling tools. Moreover, we have also analyzed the sensitivity of the best performing tools to detect experimentally validated TIPs in *Drosophila* and humans. Our results evidence that tool performance is in general lower than estimated by previous simulations, and highly variable depending on sequencing coverage and TE type. Also, we show that an appropriate combination of tools can increase the sensitivity of predictions while maintaining high precision levels.

## Results

### Tools selected for benchmarking

We selected 12 of the most widely used tools for the detection of TIPs (Table 1). Among them, four were specifically designed to detect non-reference insertions (not present in the reference genome) (RelocaTE2 [11], Jitterbug [21], Retroseq [27] and ITIS [24]), and eight were able to detect reference (present in the reference genome) and non-reference insertions (MELT [18], Popoolation TE2 [29], Teflon [1], Trackposon [7], TEMP [48], TE-locate [37], Popoolation TE [30], and ngs_te_mapper [32]. Tools specifically designed to detect presence/absence of reference TE insertions in re-sequenced genomes (i.e.: T-lex 3) [3] were not benchmarked here.

In addition to their different targets, some of the tools were family-specific (meaning that they run with one TE family at a time only), whereas most of them are able to detect insertions from all the families in the same run (broad-spectrum). Five out of the 12 tested tools were run as components of McClintock, a pipeline that combines the use of several TIP detection tools and standardizes their outputs into the commonly used BED format (Table 1).

The first difficulty that the user has to face is properly installing and making the tools run, often in a computer cluster. This can be sometimes complex due to the number of different dependencies, and especially due to the specificity of the input file preparation. In this regard, we found that RelocaTE2, PopoolationTE2 and Trackposon were the less problematic tools (Table 1). One possibility that would make the installation of these tools much easier would be to have them integrated in an environment such as Conda. This is a possibility that future developers should take into account.

### LTR-retrotransposon and MITE landscape in Nipponbare and MH63 genomes

In order to perform a benchmarking exercise that could be representative of as much as possible TIP detection in eukaryotes, we decided to use rice as a model as it has a genome of 430 Mb, which is relatively large and complex in terms of TE landscape, and that has already been considered as being as close as possible to a representative genome for angiosperms [7]. Moreover, there are several good-quality assemblies and short-read datasets of rice varieties available [23, 47]. In terms of the TEs to be detected, we concentrated on LTR-retrotransposons and MITEs as, in addition to be the most prevalent TE types in plant genomes, they are functionally and structurally very different. Indeed, whereas LTR-retrotransposons are relatively long elements (typically several Kb-long) and contain many structural features relatively easy to detect (e.g.: long LTRs at their extremities, coding capacity for several well conserved enzymatic activities), MITEs are short (typically 100–800 nt), are non-coding and do not contain structural features (except for short inverted-repeats in most cases) allowing for structural detection.

**Fig. 1:**
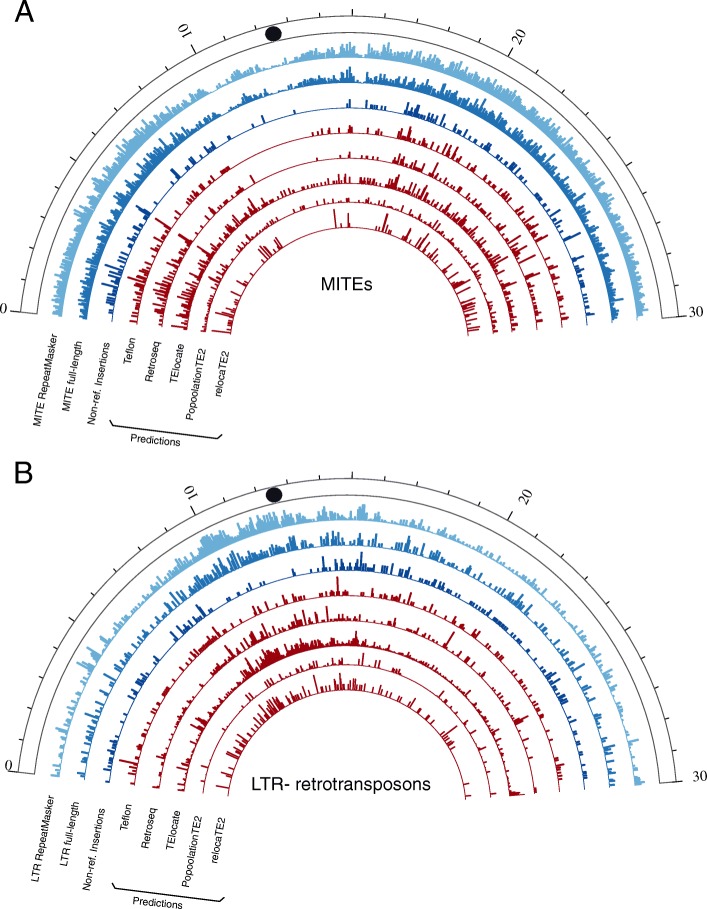
Density of MITEs (**a**) and LTR-retrotransposons (**b**) along the rice chromosome 5 (window size = 50 Kb). Black circles represent centromeres. Track 1 shows the density of all elements annotated in the chromosome by RepeatMasker. Track 2 shows the density of full-length elements. Track 3 shows the density of validated non-reference insertions (MH63-specific insertions) in the benchmarking standard. Tracks 4–8 show the density of non-reference predictions of five tools

We used a combination of structural and homology-based approaches to annotate a high-quality dataset of 3733 and 3787 full-length LTR-retrotransposons in Nipponbare and MH63 (Minghui 63) assemblies, respectively (Table 2). These elements contain intact Target Site Duplications (TSDs), Long Terminal Repeats as well as coding domains. All of them were clustered at 80% similarity over 80% length to obtain families and we derived a consensus for each family. RepeatMasker was then run with such consensuses to identify all the LTR-retrotransposon copies of the genome (including fragments and non-autonomous elements) related to the characterized families. A similar strategy was used to identify ~ 46,000 full-length MITEs, as well as ~ 200,000 partial MITE copies (see methods section). Whereas full-length LTR-retrotransposons represent a very small proportion of the total number of LTR-retrotransposon copies detected, (3%, Table 2), full-length MITEs represent an important fraction (23%). The distribution along the chromosomes of the two transposon groups is also different, with LTR-retrotransposons being more abundant in the centromeric and pericentromeric regions and MITEs populating evenly the rest of the chromosome (Fig. 1).

**Table 2:** Annotation of LTR-retrotransposons and MITEs in rice assemblies

TE Classification	Nipponbare	MH63
LTR-all ^a^	131,905	117,362
LTR full-length ^b^	3733	3787
LTR- Gypsy	1354	1303
LTR- Copia	944	759
LTR- Unclassified ^c^	1435	1725
MITE-all ^(1)^	211,732	191,113
MITE full-length ^d^	45,963	46,725

^a^ Repeatmasker fragments. Includes both intact and truncated elements

^b^ High confidence elements containing intact LTRs, TSDs and coding domains

^c^ Intact elements whose poor coding domain conservation doesn’t allow proper classification

^d^ Elements spanning more than 80% of its family consensus length

### Annotation of standard transposon insertion datasets for tool benchmarking

**Fig. 2:**
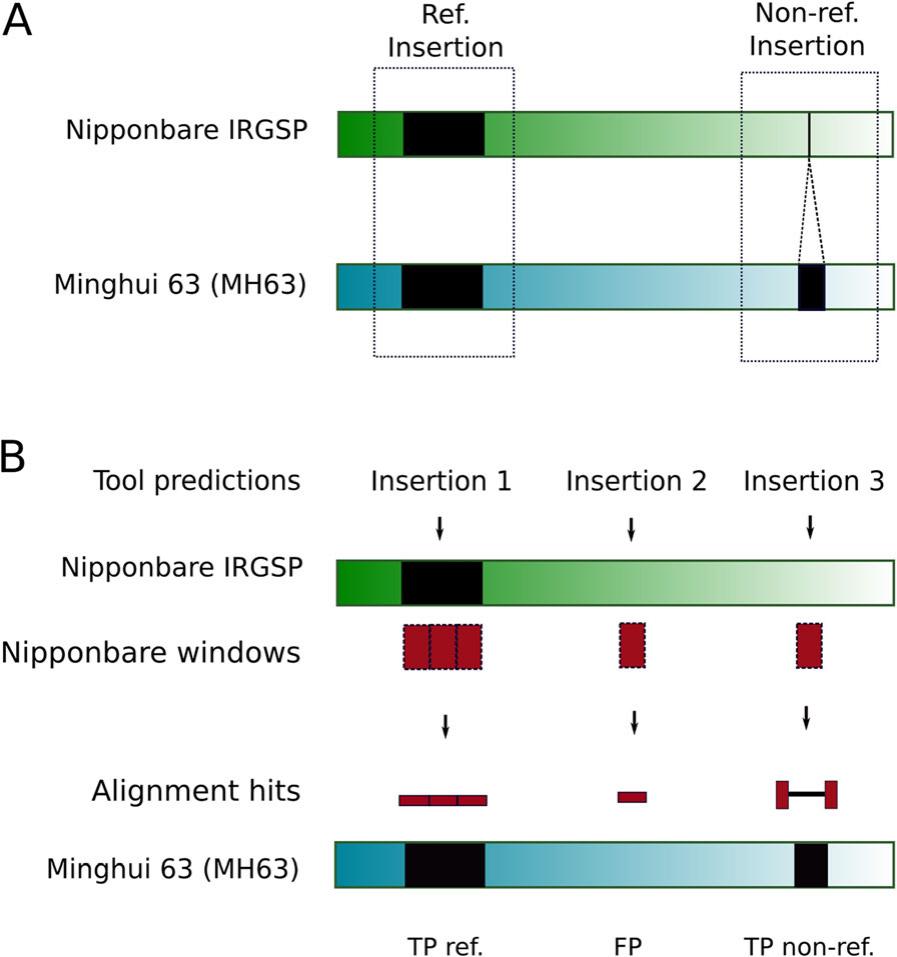
Individual validation of predicted insertions. Black boxes represent TE annotations in Nipponbare IRGSP (green rectangle) and MH63 (blue rectangle) assembled genomes. Examples of shared (reference) and MH63-specific (non-reference) insertions are shown in **a**. Insertions predicted by each tool (shown as arrows in **b**) were intersected with windows of 500 bp spanning the entire Nipponbare IRGSP genome, and windows having an intersection (red boxes, **b**) were aligned to MH63 genome. True positive reference insertions (TP ref.) were those having full-length alignments with an MH63 region where a MITE or LTR-retrotransposon was annotated. False positives (FP) have high-quality alignments (MAQ > 30) to regions were no MITE or LTR-retrotransposon was present. True positive non-reference insertions (TP non-ref) alignments were those having a spliced alignment in which the two hits were separated by a region that overlaps with a MITE or LTR-retrotransposon annotated in MH63

The most straightforward way of identifying an insertion polymorphism “in silico” when two high quality assembled genomes are available (as it is here the case), is by aligning orthologous loci. To identify the Nipponbare orthologous loci to those that in MH63 contain a TE insertion, we mapped the flanking regions of each MH63 full-length LTR-retrotransposon and MITE insertion against the Nipponbare genome. As sequence diversity and structural differences between the two genomes may complicate this analysis, we tested different flanking sequence lengths and found that 500 nt was the one that allow to identify more reference and non-reference insertions (Additional file 6: Figure S1). By inspecting the distance between the two mapped flanks, we could assign the orthology status to the locus (ie, empty site or full site). Using this approach, we were able to assign an orthology status to 86% of the MITE loci, but only to 41% of the LTR-retrotransposons loci. This was probably due to the difficulty to identify the orthologous loci of insertions siting in repetitive sequences, which is much more frequent for LTR-retrotransposons than for MITEs. Therefore, although this strategy seems the more straightforward, it has clear limitations. Moreover, as defining the precise TE-genome junctions for non-full length elements (ie, degenerated or partial elements, which are the vast majority of LTR-retrotransposons, Table 1) is challenging, we could not use this strategy to analyze the possible polymorphisms arising from non-full-length LTR-retrotransposons. To overcome those limitations and increase the dataset of curated insertions, we developed a strategy aimed at complementing the TIPs dataset with TIPs predicted with the 12 tools analyzed here (Table 2), which were individually validated. To this end we ran the different TIP-prediction tools using MH63 paired-end reads mapped to the Nipponbare reference genome. We divided the Nipponbare genome in 500 nt windows and mapped the windows containing predicted insertions (red boxes, Fig. 2) to the MH63 genome. An inspection of the aligned sections allowed determining whether the predicted insertion corresponded to a reference (shared) or non-reference (MH63 specific) insertion or if it should be considered a false positive (Fig. 2b). Indeed, in case of reference (shared) insertions, the Nipponbare and the corresponding MH63 sequences would perfectly align, showing that the sequence, which contains a TE insertion is conserved in both genomes (Fig. 2b, left); in case of a non-reference (MH63 specific) insertion, the alignment will be split by an insertion in the MH63 sequence corresponding to an annotated TE (Fig. 2b, right); and in case where the two sequences show a continuous alignment in the absence of an annotated TE insertion in Nipponbare, this will indicate that the TE prediction is a false positive (Fig. 2b, middle). After running all tools, adjacent windows corresponding to TIP predictions of the same category were merged to produce a final dataset. LTR-retrotransposon insertions are frequently more complex than MITEs (i.e.: length, tendency to form nested insertions and extremely high amount of truncated and degenerated elements, Table 2). Because of this, it was difficult in many cases to automatically validate the insertions. Therefore, manual inspection of the alignments of LTR-retrotransposons TIPs was performed, and we decided to restrict the dataset of LTR-retrotransposons to a single chromosome (chr5).

This strategy combined the power of detection of read-based methods (useful for uncovering polymorphisms derived from both full and degenerated elements), with the reliability of the validation based on alignments between high-quality assembled genomes. By using this combined approach, we increased the number of validated non-reference MITE insertions from 1898 to 3117 whereas for LTR-retrotransposons (chr5) the amount of non-reference insertions in our validated dataset increased from 22 to 239 (Additional file 2: Table S1). The result was a high-quality dataset of True Positive (TP) and False Positive (FP) reference and non-reference insertions (Additional file 2: Table S1). In addition, there were predicted insertions that did not match neither with TP nor FP (i.e.: cases that did not fit in the scenarios described in Fig. 2b). We analyzed the specific cases of unclassified non-reference insertions and found that 86% of these LTR-retrotransposon predicted TIPs and 92% of such MITE TIPs overlapped with other transposons annotated in the reference. These cases were not used for downstream analyses, as most tools specifically indicate in their manuals that they cannot properly detect nested insertions. In order to evaluate the performance of each tool, we intersected the windows corresponding to the TE insertions predicted by the tool (both reference and non-reference TE insertions) with those of the curated dataset to identify TP and FP (Fig. 2b). Insertions present in the curated dataset of TE insertions that were not detected by the evaluated tool were counted as False Negatives (FN).

Most of the tools analyzed here are able to detect insertions from all the families in the same run (broad-spectrum). Some of these tools are able to detect reference and non-reference insertions, whereas others only detect non-reference insertions. The programs use different strategies to identify these two types of insertions, and consequently we analyzed their performance separately.

### Detection of reference insertions by broad-spectrum tools

**Fig. 3:**
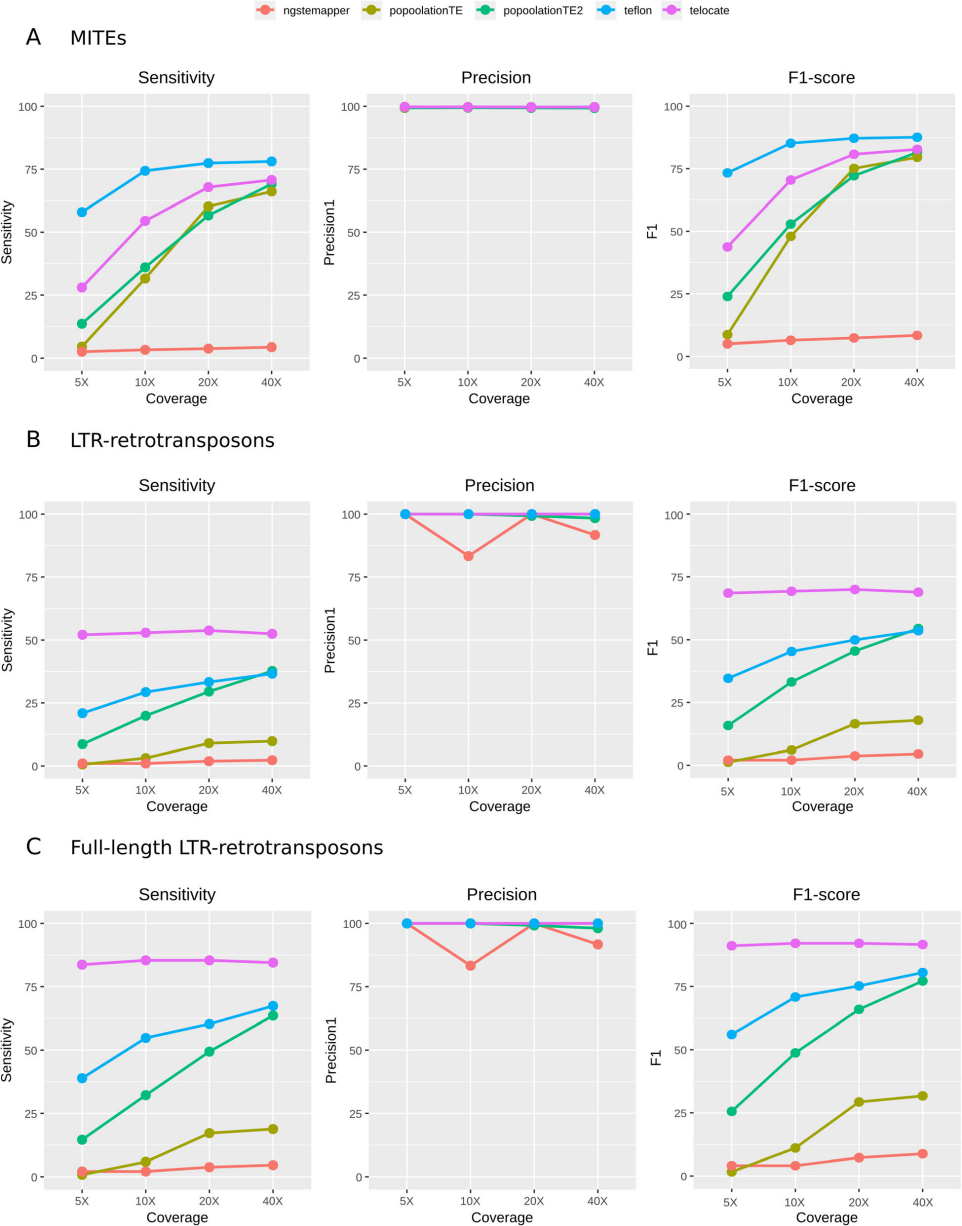
Performance of broad-spectrum tools in the detection of reference insertions of MITEs (**a**), all LTR-retrotransposons (**b**) and full-length LTR-retrotransposons (**c**)

We observed that whereas the precision detecting MITE and LTR-retrotransposon reference insertions was very high for both types of elements, the sensitivity levels of most of the tools were much higher for MITEs (Fig. 3). For MITEs, the sensitivity of most tools increased with coverage and tended to stabilize at 20-40X coverage (Fig. 3a). Teflon had consistently the best sensitivity and overall performance (F1-score) in the detection of reference MITE insertions even at low coverage, reaching a sensitivity of 74% at 10X with an almost 100% precision (Fig. 3a). All tools showed precision levels higher than 99% at all coverages, and all tools except ngs_te_mapper yielded a sensitivity higher than 60% at 40X (Fig. 3a, Additional file 3: Table S2). By contrast, the sensitivity at 5X was low in general, with Teflon being the only tool reaching more than 50% (Fig. 3a).

Regarding the detection of reference LTR-retrotransposons, the general tool performance was much lower than for MITEs (Fig. 3b). In this case, TE-locate reached the maximum sensitivity followed by Teflon and was only slightly higher than 50% (Fig. 3b), and the other tools remained below 40% sensitivity. The sensitivity of TE-locate was higher than 50% in all the coverages, whereas in Teflon, PopoolationTE2 and PopoolationTE it increased with coverage (Fig. 3b). When we focused only on the detection of full-length LTR-retrotransposons, the performance of all tools increased considerably, reaching a maximum sensitivity of 85.4% (Fig. 3c). TE-locate was again the best performer showing a sensitivity over 80% for all the coverages. We excluded the predictions of TEMP for reference insertions, as this tool is based on the detection of absences assuming the presence as default, which leads to an overestimation of the number of insertions, especially at a very low coverage.

### Detection of non-reference insertions by broad-spectrum tools

**Fig. 4:**
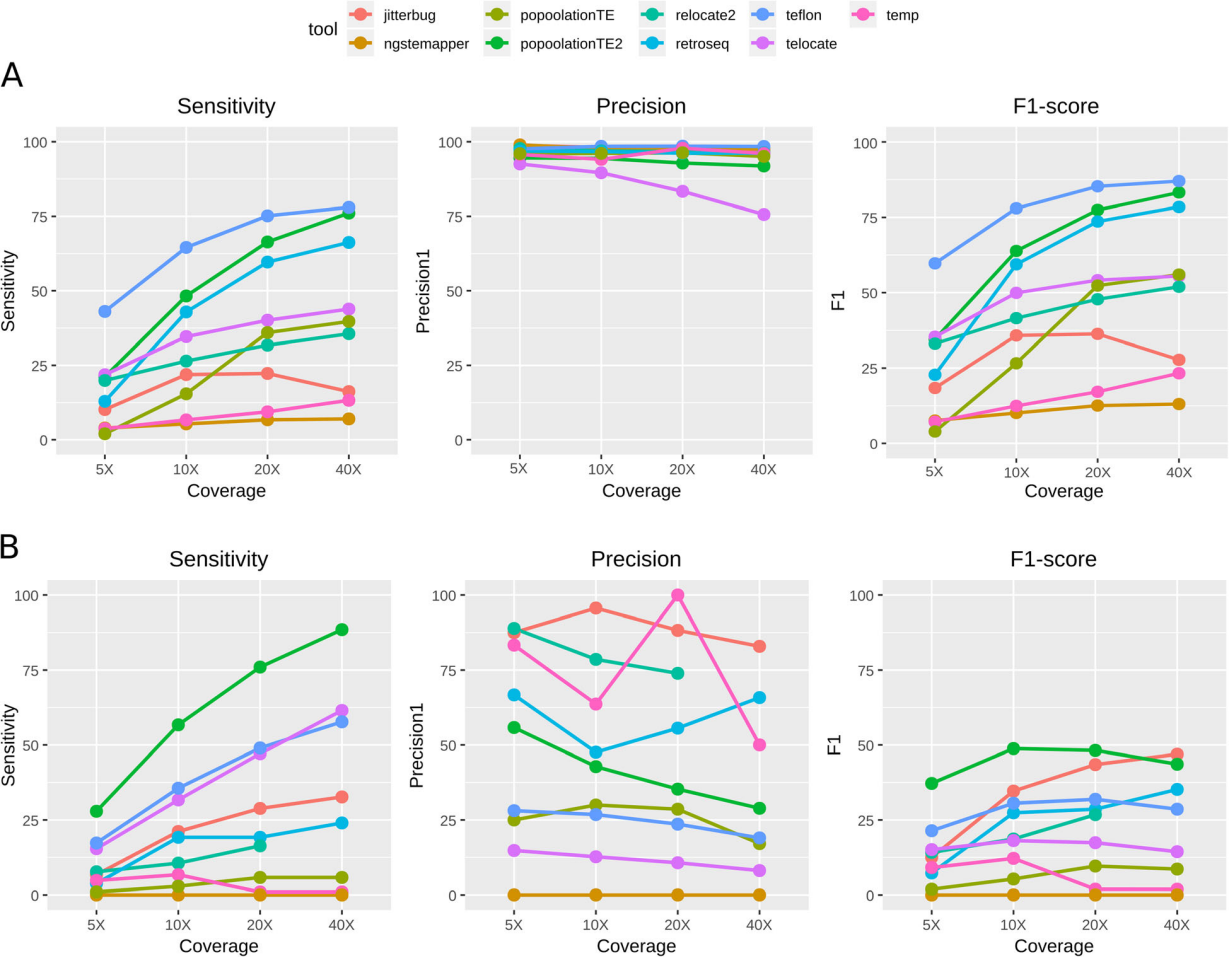
Performance of broad-spectrum tools in the detection of non-reference insertions of MITEs (**a**) and LTR-retrotransposons (**b**). Relocate2 on LTR-retrotransposons at 40X was killed after 5 days running with 8 CPUs and 64GB of RAM

All the benchmarked tools are able to detect non-reference insertions, a task that is more challenging than detecting reference insertions, as the former are not present in the reference genome to which the reads are mapped. In this case sensitivity was strongly dependent on coverage (Fig. 4). Precision was very different for MITE and LTR-retrotransposon predictions, showing a tendency to decrease at high coverage (Fig. 4). Regarding MITEs, Teflon was the best performer followed by PoPoolationTE2 and Retroseq (Fig. 4a). These tools reached a sensitivity close to 75% (up to 75.6% in 40X coverage for Teflon), whereas the rest of the tools had a much lower sensitivity (Fig. 4a). The precision was very high (> 95%) for most tools with the exception of TE-locate, which dropped from 92.5% in 5X to 75.6% in 40X. All the tools improved their performance when the coverage increased (except Jitterbug, which performed the best at 20X), with PopoolationTE2 and Retroseq showing the steepest increase, especially between 5X and 20X (Fig. 4a).

Regarding LTR-retrotransposons, PopoolationTE2 achieved the highest sensitivity, reaching a maximum of 88.5% at 40X (Fig. 4b). Nevertheless, these tools yielded a high number of false positives, which translates into low precision levels (Fig. 4b). In general, the precision detecting LTR-retrotransposons with respect to MITEs was much lower for all tools. Jitterbug was the only program with a moderate precision (> 75%) across all coverage levels, although its sensitivity was low (maximum of 32.7% at 40X) (Fig. 4b). According to the F1-score, PopoolationTE2 and Teflon were the best performers at low coverages (5X-10X), whereas at higher coverages PopoolationTE2 and Jitterbug showed the best balance between sensitivity and precision (Fig. 4b). Differently to what we previously did for reference insertions, we did not compute the performance of the tools using only full-length LTR-retrotransposons because they represent only a small fraction of the non-reference annotated insertions.

The output of most tools contains information that can be used for filtering the putative insertions to achieve more precise detection levels. We checked different filters for each program looking for gains in precision with a low cost in sensitivity. In some cases, such as Jitterbug, the precision was already very high, and the filtering was not needed. In others, the cost in sensitivity was too high and the filtering was not considered useful. For the two best-performing tools, PopoolationTE2 and Teflon, filtering did result in significative gains in precision without an excessive cost in sensitivity. For PopoolationTE2 we applied a zygosity filter of 0.7 (based on the fraction of reads supporting the insertion) which led to a drop of sensitivity for both MITEs (from 76 to 63%) and LTR-retrotransposons detection (from 88 to 65%, Additional file 7: Figure S2), but with an increase of precision, which was particularly striking for LTR-retrotransposons (from 28.9 to 91.9% at 40X). For Teflon, a zygosity filter of 1 resulted in a drop of sensitivity for MITEs (from 78 to 61.5%) and LTR-retrotransposons (from 57.7 to 44.2%) but with important gain in precision for LTR-retrotransposons (from 15.2 to 70.8%), which was not significative for MITEs (98.4 to 98.5%) (not shown). In summary, based on the F1-score, filtering by zygosity greatly improved the overall performance of PopoolationTE2 and Teflon for LTR-retrotransposon detection, whereas the effect of this filter on MITEs detection was much less pronounced due to the already high precision of the unfiltered results.

### Detection of non-reference insertions by family-specific tools

**Fig. 5:**
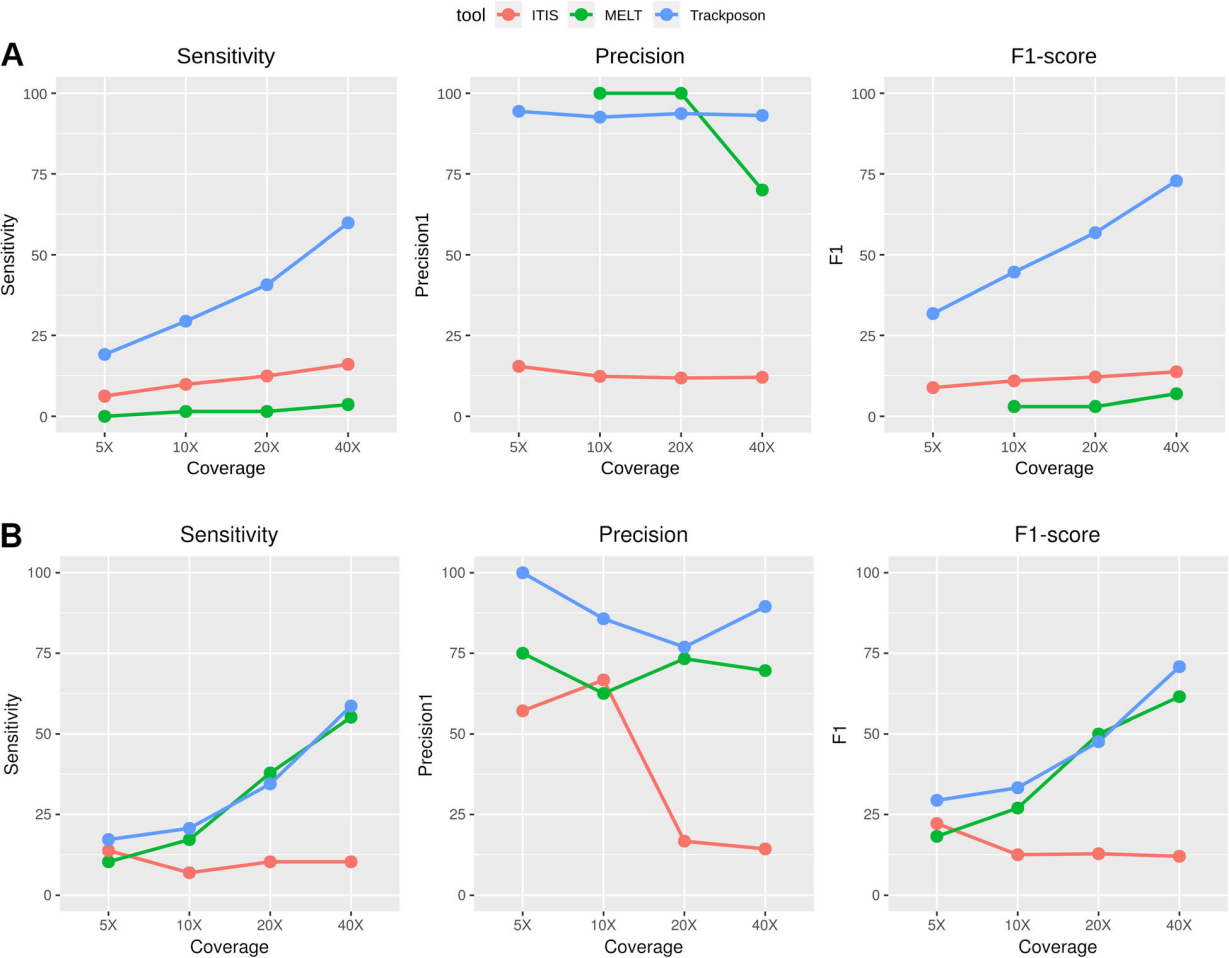
Performance of family-specific tools in the detection of non-reference insertions of MITEs (**a**) and LTR-retrotransposons (**b**). Trackposon was run on 10 kb for LTR-retrotransposons windows as described in [7]

Some tools have been designed to look only for TIPs of a single TE family instead of all families at the same time (i.e., ITIS, MELT and Trackposon). In order to analyze the performance of such tools, we used the largest MITE and LTR-retrotransposon families, which contain 194 (whole genome) and 22 (chr5) MH63-specific insertions, respectively (Additional file 7: Table S1). The analysis of MITE TIPs showed that ITIS and MELT did not perform well and displayed low sensitivity and overall F1-score levels (Fig. 5a). By contrast, Trackposon performed well, displaying up to 72.8% sensitivity with 93.1 precision at 40X coverage. In line with the results found for broad-spectrum tools, sensitivity in the detection of LTR-retrotransposons was strongly dependent on the coverage. Trackposon and MELT showed moderate sensitivity levels at 40X (58.6 and 55.2%, respectively) whereas ITIS reached a maximum of sensitivity of 13.8%. Regarding precision, Trackposon was the best performer with values ranging between 76.9 and 100% (Fig. 5b).

### Overlap between TIP prediction tools

**Fig. 6:**
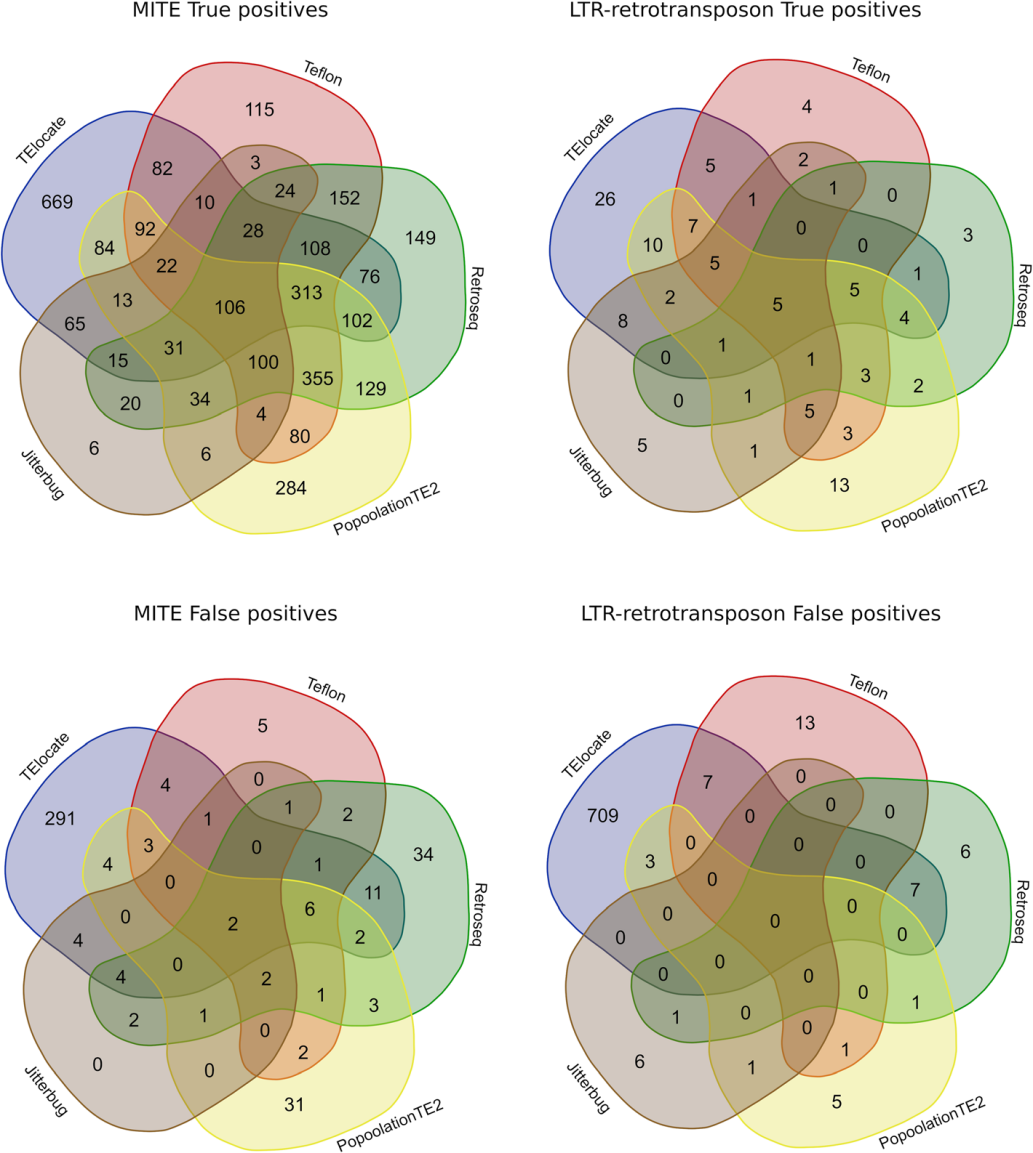
Venn diagrams representing the detection overlap in non-reference true positives and false positives for MITEs and LTR-retrotransposons

As there is no tool showing 100% sensitivity, we asked whether the predictions of the different tools were common or specific for each tool. We evaluated the overlap of the detected non-reference true and false positives for the five better performing tools for MITE or LTR-retrotransposon TIP predictions (40X), taking into account their sensitivity and precision. In spite of the difference in the amount of predictions between MITEs and LTR-retrotransposons, the results showed very similar trends: 54% of TP were detected only by one tool for both MITE and LTR-retrotransposon insertions (Fig. 6). As expected, the FP detected were tool-specific in the vast majority of the cases (90.2% were detected by only one tool for MITEs and 98% for LTR-retrotransposons). The number of insertions detected by all tools was very low (1.3% of all TIPs detected for MITEs and 1.4% for LTR-retrotransposons). These results suggest that combining tools may increase the sensitivity of the TIP detection, although this may come with the cost of decreasing precision, as false positives are highly tool-specific.

### Combining tools to improve sensitivity

**Fig. 7:**
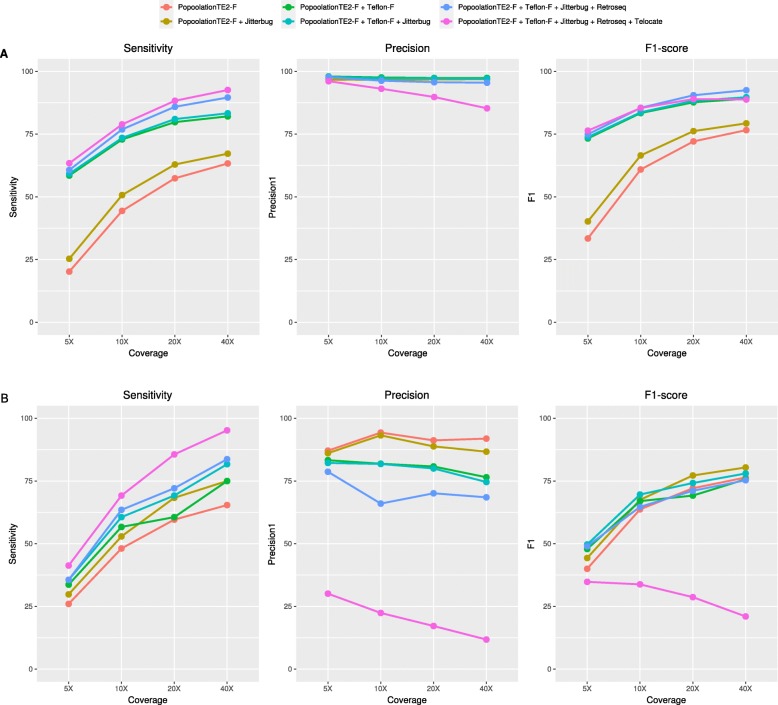
Performance of tool combinations in the detection of non-reference insertions in MITEs (**a**) and LTR-retrotransposons (**b**)

Our previous results suggest that a combination of tools could be useful to increase the sensitivity in identifying non-reference transposon insertions. To this end, we combined the predictions of PopoolationTE2 (the overall best performer) sequentially with up to four tools selected based on their sensitivity and/or precision levels. As a general trend, the combination of tools led to higher sensitivity levels, reaching more than 90% for both MITEs and LTR-retrotransposons at 40X coverage when combining five different tools (Fig. 7). However, the increase in sensitivity comes with a decrease in precision, particularly clear for LTR-retrotransposons, that approaches 10% for 40X coverage when combining five different tools. The results presented suggest that the combination of two tools provided the best balance between sensitivity and precision. Specifically, the combination of zygosity-filtered PopoolationTE2 and Teflon for MITEs reached 82.1% sensitivity and 97.4% precision at 40X. Regarding LTR-retrotransposons, the combination of zygosity-filtered PopoolationTE2 and Jitterbug reached 75% sensitivity and 86.7% precision at 40X.

**Fig. 8:**
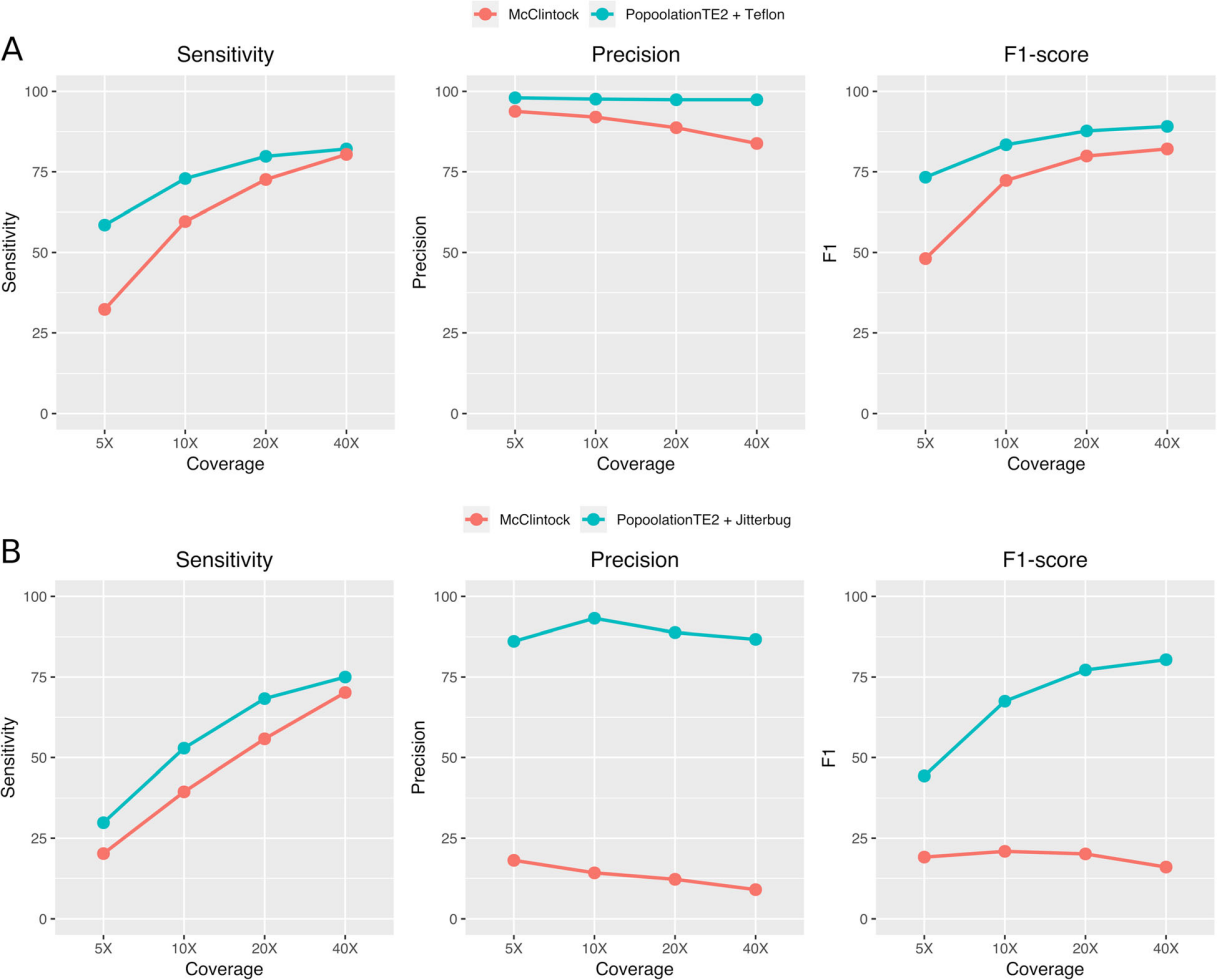
Performance comparison between McClintock pipeline and our proposed tool combinations for MITEs (**a**) and LTR-retrotransposons (**b**). PoPoolationTE2 and Teflon are filtered by zygosity as explained in the text (cutoffs of 0.7 and 1, respectively)

As already mentioned, McClintock is an available pipeline that combines several tools. Therefore, we compared the performance of the combination of tools here proposed with that of the McClintock pipeline, which combines the use of Retroseq, TEMP, TE-locate, PopoolationTE and ngs_te_mapper (we excluded RelocaTE from the pipeline due to excessive running time). The combination of tools here proposed (PopoolationTE2 and Jitterbug for LTR-retrotransposon insertions and PoPoolationTE2 and Teflon for MITEs) yielded consistently a better sensitivity and much better precision and F1-scores than McClintock at all coverages (especially in the case of LTR-retrotransposons, Fig. 8). The most important differences were found in precision at intermediate and high coverages. As an example, for MITEs at 40X PopoolationTE2-Teflon had 97.4% precision whereas McClintock had 83.8% (Fig. 8a). Regarding LTR-retrotransposons at 40X, PoPoolationTE2-Jitterbug precision was 86.7%, whereas that of McClintock dropped to 9% (Fig. 8b).

### Evaluation of best-performing tools using *Drosophila* and human datasets

In order to evaluate whether the benchmarking results using rice data could be extrapolated to data obtained from other species, we benchmarked the best performing tools (PoPoolationTE2, Teflon and Jitterbug) using PCR-validated TIPs from *Drosophila* and humans. The *Drosophila* dataset consisted of 81 TIPs from ten *Drosophila* lines sequenced at an average coverage of 42X [22]. This dataset contained TIPs from 12 different transposon families, including retrotransposons (LTR and LINE) and cut-and-paste DNA transposons (TIR) experimentally validated by Lerat et al. [31] Merenciano et al. [33] and Ullastres et al. [46] (Additional file 4: Table S3). The human dataset consisted of 148 TIPs obtained from one human individual at a coverage of 20X [44]. This dataset consisted of TIPs related to ALU, SVA and LINE-1 retroelements. In the analysis of human insertions, we also included MELT, as it is the best-established tool for the detection of human TE polymorphisms. The detection levels of PoPoolationTE2 and Teflon in *Drosophila* were moderately high (69.1% of the insertions, Table 3 and Additional file 5: Table S4), and substantially higher than Jitterbug (44.4% of the insertions). Using the combination of the three tools, we were able to detect 81.5% of the insertions. These results are in high concordance with the sensitivity levels found using rice data with LTR-retrotransposons and MITEs, where PoPoolationTE2 and Teflon showed superior detection levels to Jitterbug (Fig. 4). Regarding the human sample, MELT was the best tool identifying homozygous insertions (97.8%, Table 4), whereas PoPoolationTE2 was the best detecting heterozygous insertions (88.2%). Taking into account both kind of insertions, PoPoolationTE2 outperformed MELT, displaying an average detection level of 90.5%. The detection rate of these two programs was higher on human data than in *Drosophila* or rice, where sensitivity levels rarely exceeded 70% using 20X coverage (Fig. 4). The detection levels of Jitterbug were similar to those found using *Drosophila* and rice, ranging from 47.8 to 51%. Teflon was unable to complete the task and the process was killed after five running days. Using the combination of tools, the detection rate increased only 3.4% for the human dataset, reaching up to 93.9% (Table 4).

**Table 3:** Number of insertions detected by PoPoolationTE2, Jitterbug and Teflon using a validated *Drosophila melanogaster* dataset

	RAL-737	RAL-40	RAL-801	RAL-802	RAL-850	RAL-502	RAL-508	RAL-491	RAL-235	RAL-21	TOTAL	%
Validated insertions	17	16	9	7	4	5	7	5	4	7	81	
PoPoolationTE2	12	5	9	5	3	3	6	5	3	5	56	69,1
Jitterbug	11	2	3	5	2	2	4	2	3	2	36	44,4
Teflon	12	6	9	4	3	4	5	4	4	5	56	69,1
Combination	15	6	9	7	3	4	7	5	4	6	66	81,5

Total number of insertions detected by each tool on each line is provided in Additional file 5: Table S4

**Table 4:** Number of insertions detected by Jitterbug, MELT and PoPoolationTE2 using a validated human dataset

Tool	Homozygous	Heterozygous	Total
Validated insertions	46	102	148
PoPoolationTE2	44 (95,7%)	90 (88,2)	134 (90,5%)
Jitterbug	22 (47,8%)	52 (51,0%)	74 (50,0%)
Teflon ^a^	–	–	–
MELT	45 (97,8%)	84 (82,4%)	129 (87,2%)
Combination	45 (97,8%)	94 (92,2%)	139 (93,9%)

^a^ Teflon was killed after 5 days running with 12 CPU and 300GB of RAM

Total number of insertions detected: PoPoolationTE2 (ref and non-ref) = 186,038; Jitterbug (non-ref) = 624; MELT (non-ref) =1297

### Running time

**Fig. 9:**
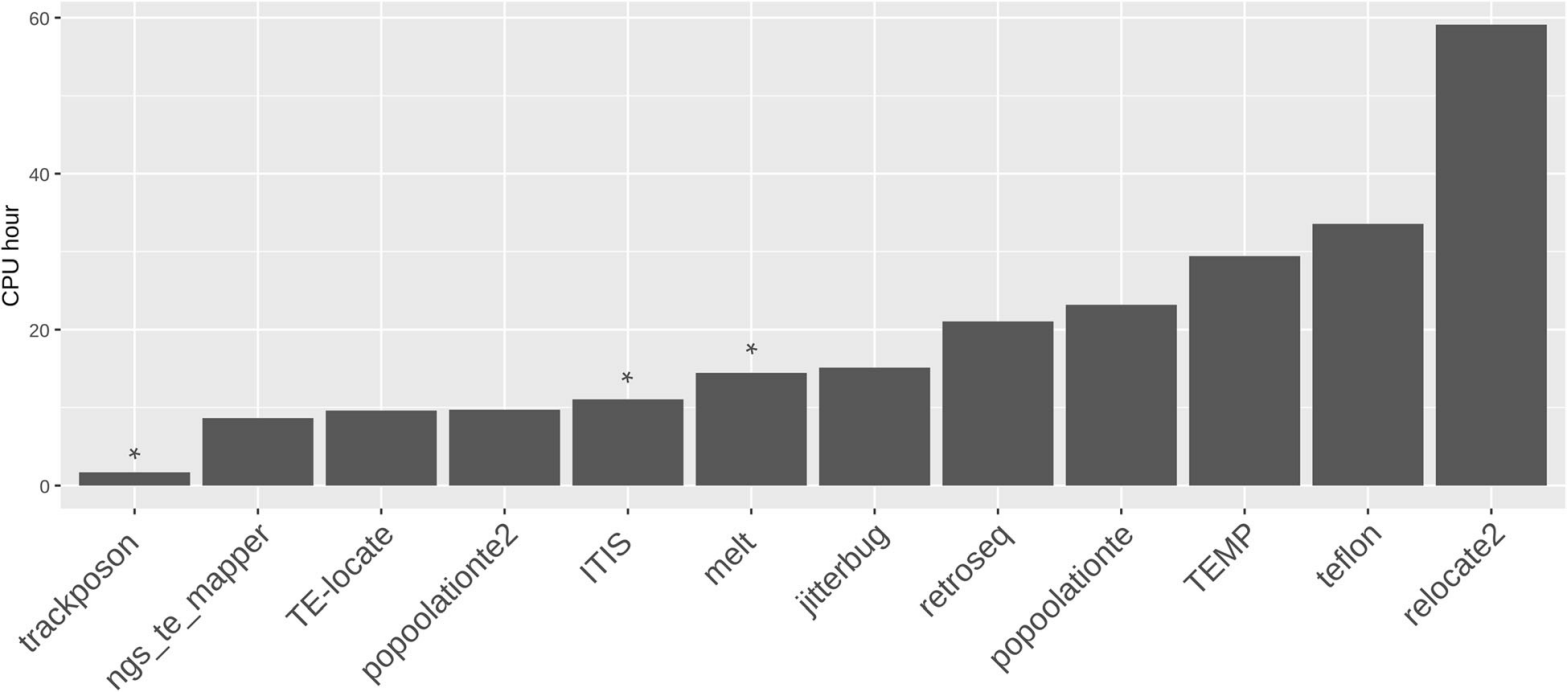
Running time of each tool to perform the detection of MITEs in a 10X dataset. Family-specific tools are marked with an asterisk. All tools were run using 8 CPUs and 64GB of RAM

Computation time is a limiting factor when running TIP detection tools in large datasets. Therefore, it is an important criterion to take into consideration for selecting the most appropriate tool for a specific experiment. We tested the time needed by the tools to finish the prediction with a 10X dataset and 432 MITE families as input. It is important to mention that three tools (Trackposon, ITIS and MELT) work on a per-family basis. In these cases, the reported time was that needed to finish the prediction for a single family. By contrast, the remaining tools work with all the annotated TE families at the same time. According to our results, Trackposon was the fastest tool, with only 1.7 CPU hours needed to finish (Fig. 9). Among the general tools, ngs_te_mapper, TE-locate and PoPoolationTE2 were the fastest tools, with 8.6, 9.6 and 9.7 CPU hours needed to finish the prediction for the 432 families. RelocaTE2 took the largest amount of time to finish the prediction (59.1 CPU hours) (Fig. 9).

## Discussion

### The use of real data is essential for an accurate benchmarking of TE insertion detection tools

There are several tools available to detect TIPs from short-read resequencing data, and some efforts have been made to validate the performance of such tools [36, 41]. However, their benchmarking has been essentially based on simulated TE insertions and simulated short reads. It is challenging to perfectly simulate sequencing errors, local coverage variations, biases due to GC content or other genome specific biases that real short-read datasets contain. Similarly, the heterogeneity of real transposon insertions, with polymorphic truncated or degenerated elements and elements inserted in highly repetitive regions, among other confounding effects, are also difficult to simulate. As a consequence, the benchmarking using simulated data may be overestimating the performance of the TIP prediction tools. Indeed, our results show that, most of the tools here analyzed have a lower sensitivity than previously reported. For example, RelocaTE2 and TEMP were previously benchmarked on simulated rice data, and the sensitivity of both tools was estimated to be higher than 99% at 10X [11]. On the contrary, our results using a dataset of real insertions and real short-read data show that both programs perform very different, with TEMP having a maximum sensitivity of only 13.3% for MITE detection and RelocateTE2 showing a 35.6% sensitivity. Similarly, we previously reported a sensitivity of close to 90% for Jitterbug, a program developed in our laboratory, using real short reads on simulated TE insertions [21]. Our results now show that for the dataset analyzed (real TIPs and real short reads) the maximal sensitivity is of 32.7% (Fig. 4, LTR-retrotransposons), although it does so with a relatively high precision. Therefore, our results suggest that the sensitivity and precision previously reported for TIPs detection tools, determined using simulated data, are probably overestimated and that the real performance of these tools is probably lower. We think that the performance levels of the different tools presented here are a much better estimation of their detection ability on real datasets. It is important to note, however, that depending on the genome to be analyzed, parameters used and especially on the quality of the annotation of the reference genome the performance of the programs may vary. All the programs benchmarked here are based on the detection of discordant paired-end reads and/or split-reads at the junction of TE insertions. Among the different confounding factors that can interfere with the detection process, the quality of the TE annotation of the reference genome and in particular of the proper definition of the TE-genome junctions, is an important one. Therefore, it is important to work on refining the annotation of the TEs (or at least the more interesting TE families for the purpose of the study) before searching for TIPs.

### Tool performance varies depending on TE family

Eukaryote genomes contain a high diversity of TE elements with very different copy numbers and functional and structural characteristics, which may impact on the ability of TIP detecting programs to reliably identify their insertions. Because of that, we decided to benchmark the different programs using two very different types of TEs that, in addition, are the most prevalent in plants: MITEs and LTR-retrotransposons. The results presented here show that, as expected, the analyzed tools do not detect different TE types with the same sensitivity and precision. MITEs and LTR-retrotransposons represent extreme examples based on their length and complexity, and the performance of the tools when used with other TEs will probably be in the range of this case-study. The analysis of the sensitivity of the best performing tools in detecting TIPs produced by different types of transposons (including LINEs, LTR-retrotransposons and cut-and paste TIR transposons) in *Drosophila* and humans suggests that this is indeed the case. Our results indicate that MITEs are detected with better sensitivity and precision than LTR-retrotransposons. The difference is especially relevant in the detection of non-reference insertions, where most tools show low precision levels for LTR-retrotransposons. In the present study, we ran all samples in default mode or using the parameters described by the authors in the corresponding manuscripts or manuals (Additional file 1). Nevertheless, we show that precision can be increased by applying specific filters to the results. For example, we show that, for some programs, LTR-retrotransposon detection can be drastically improved by applying a zygosity filtering. Applying such filtering may be a good strategy when not intending to study somatic insertions which should in most cases be heterozygous. The difficulties of detecting LTR-retrotransposons come from the complexity of the elements and from the local regions where they insert. It is known that LTR-retrotransposons (especially those of the Gypsy superfamily) tend to integrate in heterochromatic regions enriched in other TEs. These repetitive regions are likely a source of false positives that affects all the programs tested. These repetitive regions are, in fact, difficult to annotate and polymorphisms within these regions may be challenging to detect even using long-read data or when aligning good-quality assemblies. By contrast, MITEs tend to integrate close to genes [25] and their flanking regions are more likely to be unique in the genome. The presence of non-repetitive TE flanks greatly simplifies the detection of TIPs, as the probability of finding multimapping reads in these regions is minimal.

Another important consideration related to the different TE families is the quality of the annotation. MITEs are easy to annotate and usually have well defined boundaries. By contrast, LTR-retrotransposons form nested insertions and are often degenerated. This makes very difficult to accurately define their boundaries, and as a consequence many chimeric elements are usually annotated. As already mentioned, an accurate TE annotation is essential to increase the capacity of the tools to identify TE insertions based on short-read data. In this context, it could be a good strategy to identify and remove chimeric transposons from the annotation prior to using any of these tools (ie, when working with consensuses or with the actual annotation). A chimeric or nested transposon, for example an LTR-retrotransposon with a MITE inserted inside, will be targeted by reads arising from the two elements, and other MITE insertions of the same family present elsewhere in the genome could be wrongly identified as LTR-retrotransposons insertions by the TIP detection tools.

### Influence of the type of genome on the performance of the tools

The ability of any of the tools to detect TIPs depend on the nature of the transposon insertion itself and its flanking genome sequence, and none of them can detect new transposon insertions in repetitive regions. Therefore, in addition to the type of transposon generating the TIP, as already discussed, the performance of the tools may depend on the genome under study. For this reason, we have analyzed the sensitivity of the tools that performed the best using rice data on *Drosophila* and human data and compared their performance on the different datasets. The sensitivity of the different programs analyzed in *Drosophila* was very similar to the one obtained in rice. As the genomes of rice and *Drosophila* are relatively different, the former being much bigger (430 Mb vs 175 Mb) and with a higher content of repetitive sequences (37% vs 20%), this suggests that the performance of the tools is relatively independent of the genome used, and that the benchmarking here presented could be of use for TIP analysis in many different systems.

This analysis also showed that the tools that performed best on rice had an even better sensitivity on human data. The difference of sensitivity was particularly clear for PoPoolationTE2 and MELT. Although this could indicate a difference of the performance of these tools in the two genomes, it could also be due to the particular nature of the human dataset. Indeed, the dataset of validated TIPs in humans contains insertions from TE families (LINE-1, ALU, SVA) that were detected in the first place using only one method, based on split-read and read-pair information [44] and therefore the sensitivity of the programs on this dataset could be overestimated. It is worth mentioning that the PCR-validated Drosophila and human insertions have been predicted using a small number of tools in the original publications, and therefore it includes only a subset of all the insertions present in these genomes. Moreover, the human and Drosophila datasets were validated by PCR, which could have introduced a bias in the TEs that were included in these datasets. However, note that the number of families included in the human and Drosophila validation datasets are similar or bigger than the ones included in the rice dataset and contain both full-length and truncated TEs.

### Sequencing coverage critically impacts TIP detection

Independently of the different performance found between TE families, we found that coverage has a major impact on tool performance for all the TE families tested. In general sensitivity increases with increasing coverage. Therefore, homogenization of sample coverage is essential when using TIPs prediction tools to quantitatively compare the transposition rates between organisms or populations. Some tools like PopoolationTE2 have internal steps to carry out this task. Nevertheless, for qualitative studies coverage homogenization is discouraged as down-sampling high-coverage datasets leads to a smaller number of detected insertions. It is important to note that the increase of sensitivity with increasing coverage comes, in most cases, with a decrease in precision. Therefore, depending on the goals of the study a different level of coverage may be suitable. From the data presented here it seems that a coverage below 20X is probably not suited for most analyses, as the probability of missing true insertions is very high.

### Strategies to increase tool performance

The fact that an important fraction of the insertions detected by the different tools are not shared supports the fact that combining different tools may increase the quality of the results [36]. However, simply increasing the number of tools does not necessarily increase the quality of predictions, due to the accumulation of tool-specific false positives (ie, the combination of five tools yielded 95% of sensitivity but only 11.8% precision in non-ref LTR-retrotransposon detection, Fig. 7). This is due to the fact that whereas many true insertions are detected by several tools, most false positives are tool-specific (Fig. 6). Combining a limited number of well-performing tools may be the best approach. Indeed, our results show that with the dataset used, the combination of PoPoolationTE2 and Jitterbug to detect LTR-retrotransposon insertions, or PoPoolationTE2 and Teflon to detect MITEs yielded superior TIP annotations (better F1-score) than the tools alone. Also, the performance of these tool combinations was better than that of the McClintock pipeline, especially regarding LTR-retrotransposons. In this sense, we recommend combining tools based on their high precision and not only on their high sensitivity (ie, PoPoolationTE2 and Jitterbug). Nevertheless, there can be situations in which sensitivity has a priority over precision (ie, re-sequencing of a single individual, or interest only on a few families). In such cases, running more tools can be an alternative and manual curation should be considered.

### Selecting the appropriate tools for detecting TE insertions in resequencing data

Depending on the objective of the analysis, a family-specific tool could be more interesting than a broad-spectrum tool. For example, when tracking the effect of certain treatment in a concrete set of elements. Another important consideration is that the amount of storage needed is smaller in comparison to broad-spectrum tools, due to the smaller size of the alignment files. For such cases, a tool such as Trackposon could be a good option due to its fast speed, moderate sensitivity and high precision. Nevertheless, as a drawback, Trackposon does not report the exact insertion point and, which could be a limitation for some studies. In those cases, MELT can be an interesting alternative, although it requires adjusting family-specific parameters to produce high-quality results. This might be indeed the cause why MELT did not perform well on the detection of rice MITEs. In general, it is possible that the tools analyzed here, which were not specifically designed for MITEs and LTR-retrotransposons, may work better for other types of TEs or with modifications in the parameters used. Based on our results, if the objective of the study is to analyze insertions of more than one family, and the storage space is not a major limitation, using some of the top broad-spectrum tools such as PoPoolationTE2 is probably a better option as those programs can also be relatively fast and show high sensitivity and precision independently of the species and TE type analyzed.

## Conclusions

Besides the important efforts of tool developers, our results suggest that the identification of TIPs is still challenging. We propose here a number of approaches, such as combining tools, which can be followed depending on the purpose of the study and the TE families to be analyzed, that can provide good results. However, it is important to note that in the best scenario (combining optimal tools at best coverage, Fig. 7) and having a good TE annotation of the reference genome, the sensitivity could be around 70% with a precision of 80–90% for non-reference insertions. These numbers may be enough for most studies, but it is important to keep in mind that some insertions will be missed, especially when estimating insertion frequencies or when using TIPs for GWAS, for example. There are major limitations like the length of the reads that can be resolved with current technologies (ie long-read sequencing) and will certainly improve in the following years. But there is still the need to develop new algorithms specifically designed to identify TIPs from long reads, to generate highly curated TE annotations of reference genomes and also more independent benchmarks on real data to evaluate the performance of tools under different conditions.

## Methods

### Sequence data used

We used the available data for the japonica Nipponbare (GCA_000005425.2) and the indica MH63 (GCA_001623365.1) assemblies, and the short-read resequencing of MH63 (SRX1639978), which were used to generate the original assembly.

### MITE annotation

MITE-hunter [19] was run on Nipponbare and MH63 assemblies to detect MITEs families, which were then combined with the high-quality predictions available in PMITE database [9] (only families carrying TSD). Clustering at 90% was performed to remove redundancy using cd-hit [17] and produce a final library. RepeatMasker (http://www.repeatmasker.org/) was run to annotate all regions having significant homology with any of the MITE families. The annotations were further screened to discriminate full-length elements (consensus length ± 20%) from truncated hits.

### LTR-retrotransposon annotation

LTR-retrotransposons were identified by running LTRharvest [14] on IRGSP and MH63 assemblies with default parameters. The internal conserved domains of these elements were obtained running hmmscan [13], and only coding elements were retained for further analyses. The identified elements were clustered with Silix [34] according to the 80–80 rule. All the elements in each family were aligned with Mafft [26] and trimmed with Trimal [6]. Consensus sequences were built from the alignments using the EMBOSS package [40].

### Determination of benchmarking standards

We took advantage of the availability of two high quality rice genome assemblies (IRGSP and MH63, the former used as reference) in order to obtain a curated dataset of real “reference” (orthologous) and “non-reference” (specific to MH63) insertions as explained in Fig. 2. Mapping of reference and non-reference windows to MH63 genome was performed using BBmap (https://sourceforge.net/projects/bbmap/). Intersections between annotations were done with BEDtools [38].

### *Drosophila* and human benchmarking datasets

The *Drosophila* dataset consisted of 81 TIPs from ten *Drosophila* lines sequenced at an average coverage of 42X [22], and validated by PCR by Lerat et al. [31], Merenciano et al. [33] and Ullastres et al. [46] (Additional file 4: Table S3). In Lerat et al. [31], TIPs were predicted using TIDAL [39] and PoPoolationTE2 [29] using 14 European *D. melanogaster* pooled populations (average coverage of 90X). Briefly, validated TIPs were present in the DGRP population and at least in one European population at > 10% frequency, not present in the Y chromosome, and with a predicted length of < 6 kb to avoid problems with PCR amplification. In Ullastres et al. [46], TIPs were predicted by TIDAL in the DGRP population [39]. Validated TIPs were inserted in regions with recombination rates > 0, and present in at least 15 DGRP strains. Finally, in Merenciano et al. [33] TIPs were also predicted by TIDAL in the DGRP population [39] and all belonged to the *roo* family. Both full-length and truncated copies were validated, as no TE length filter was applied.

The human dataset consisted of 148 TIPs obtained from a human individual (NA12891, SRA accession SRX207113) [44]. Original sequencing coverage of the human genome was down sampled to 20X.

### TIP prediction

Predictions of transposon insertions were done using the 12 tools shown in Table 2 using the default parameters and / or following the recommendations of the authors. The scripts used for running each of the tools are shown in Additional file 1.

### Evaluation parameters

We used the following parameters for evaluating the ability of each tool to detect MITEs and LTR-retrotransposons: True positives (TP): Insertions detected by any tool matching with our curated dataset of TPs. False positives (FP): Insertions detected by any tool matching with our curated dataset of FPs. False negatives (FN): Insertions present in our curated dataset of TPs, not detected by the evaluated tool. These primary parameters were used for calculating the final benchmarking ratios that have been previously used for assessing the performance of similar tools [41].

- ♦ Sensitivity = TP/ (TP+ FN).
- ♦ Precision = TP/ (TP + FP)
- ♦ F1-score = 2 x [(Precision x Sensitivity) / (Precision + Sensitivity)]

## Supplementary information

**Additional file 1.** Scripts used to run all TIP detection tools. (.sh) (SH 13 kb)

**Additional file 2 : Table S1.** Insertion dataset used for benchmarking. Contains all the TP reference and non-reference windows, as well as all FP windows. (.xlsx)

**Additional file 3 : Table S2.** Numerical benchmark results. (.xlsx)

**Additional file 4 : Table S3.** *Drosophila melanogaster* TE insertions validated by PCR. TE names are provided in the table when authors gave names to the non-reference insertions. All insertions were validated based on PCR band sizes. Validated insertion sites are provided when authors sequenced PCR bands evidencing the presence of a particular insertion. ND: not determined. (xlsx)

**Additional file 5 : Table S4.** Total number of insertions detected by PoPoolationTE2, Jitterbug and Teflon in ten Drosophila lines. (xlsx)

**Additional file 6 : Figure S1.** Number of MH63 reference and non-reference insertions detected by direct comparison of 1000 LTR-retrotransposon flanking sites of different sizes from MH63 and Nipponbare genomes. (.pdf)

**Additional file 7 : Figure S2.** Application of zygosity filtering to PoPoolationTE2. PoPoolationTE2-F means that it was run and filtered at zygosity 0.7. PopoolationTE2-R corresponds to the raw results. (.png)

## Data Availability

The datasets analyzed during the current study are available in the NCBI repository: - Nipponbare Assembly: GCA_000005425.2 - MH63 assembly: GCA_001623365.1 - Short-read resequencing data of MH63: SRX1639978 - Human resequencing reads: SRX207113 - *Drosophila* resequencing reads: PRJNA36679

## References

[1] Adrion JR, Song MJ, Schrider DR, Hahn MW, Schaack S (2017). Genome-wide estimates of transposable element insertion and deletion rates in Drosophila melanogaster. Genome Biol Evol.

[2] Alkan C, Coe BP, Eichler EE (2011). Genome structural variation discovery and genotyping. Nat Rev Genet.

[3] Bogaerts-Márquez M, Barrón MG, Fiston-Lavier A-S, et al. T-lex3: an accurate tool to genotype and estimate population frequencies of transposable elements using the latest short-read whole genome sequencing data. Bioinformatics. 2019, btz727. 10.1093/bioinformatics/btz727 PMC7703783 31580402

[4] Butelli E, Licciardello C, Zhang Y (2012). Retrotransposons control fruit-specific, cold-dependent accumulation of anthocyanins in blood oranges. Plant Cell.

[5] Cao Y, Chen G, Wu G (2019). Widespread roles of enhancer-like transposable elements in cell identity and long-range genomic interactions. Genome Res.

[6] Capella-Gutiérrez S, Silla-Martínez JM, Gabaldón T (2009). trimAl: a tool for automated alignment trimming in large-scale phylogenetic analyses. Bioinformatics.

[7] Carpentier M-C, Manfroi E, Wei F-J (2019). Retrotranspositional landscape of Asian rice revealed by 3000 genomes4. Nat Commun.

[8] Carr M, Bensasson D, Bergman CM (2012). Evolutionary genomics of transposable elements in Saccharomyces cerevisiae. Plos One.

[9] Chen J, Hu Q, Zhang Y, Lu C, Kuang H (2014). P-MITE: a database for plant miniature inverted-repeat transposable elements. Nucleic Acids Res.

[10] Chen J, Lu L, Benjamin J (2019). Tracking the origin of two genetic components associated with transposable element bursts in domesticated rice. Nat Commun.

[11] Chen J, Wrightsman TR, Wessler SR, Stajich JE (2017). RelocaTE2: a high resolution transposable element insertion site mapping tool for population resequencing. PeerJ.

[12] Chuong EB, Elde NC, Feschotte C (2017). Regulatory activities of transposable elements: from conflicts to benefits. *Nature Reviews*. Genetics.

[13] Eddy SR (2011). Accelerated profile HMM searches. PLoS Comput Biol.

[14] Ellinghaus D, Kurtz S, Willhoeft U (2008). LTRharvest, an efficient and flexible software for de novo detection of LTR retrotransposons. BMC Bioinformatics.

[15] Ewing AD (2015). Transposable element detection from whole genome sequence data. Mob DNA.

[16] Flutre T, Duprat E, Feuillet C, Quesneville H (2011). Considering transposable element diversification in de novo annotation approaches. Plos One.

[17] Fu L, Niu B, Zhu Z, Wu S, Li W (2012). CD-HIT: accelerated for clustering the next-generation sequencing data. Bioinformatics.

[18] Gardner EJ, Lam VK, Harris DN (2017). The Mobile element locator tool (MELT): population-scale mobile element discovery and biology. Genome Res.

[19] Han Y, Wessler SR (2010). MITE-hunter: a program for discovering miniature inverted-repeat transposable elements from genomic sequences. Nucleic Acids Res.

[20] Hénaff E, Vives C, Desvoyes B (2014). Extensive amplification of the E2F transcription factor binding sites by transposons during evolution of Brassica species. Plant J.

[21] Hénaff E, Zapata L, Casacuberta JM, Ossowski S (2015). Jitterbug: somatic and germline transposon insertion detection at single-nucleotide resolution. BMC Genomics.

[22] Huang W, Massouras A, Inoue Y (2014). Natural variation in genome architecture among 205 Drosophila melanogaster genetic reference panel lines. Genome Res.

[23] International Rice Genome Sequencing Project (2005). The map-based sequence of the rice genome. Nature.

[24] Jiang C, Chen C, Huang Z, Liu R, Verdier J (2015). ITIS, a bioinformatics tool for accurate identification of transposon insertion sites using next-generation sequencing data. BMC Bioinformatics.

[25] Jiang N, Wessler SR (2001). Insertion preference of maize and rice miniature inverted repeat transposable elements as revealed by the analysis of nested elements. Plant Cell.

[26] Katoh K, Standley DM (2013). MAFFT multiple sequence alignment software version 7: improvements in performance and usability. Mol Biol Evol.

[27] Keane TM, Wong K, Adams DJ (2013). RetroSeq: transposable element discovery from next-generation sequencing data. Bioinformatics.

[28] Kobayashi S, Goto-Yamamoto N, Hirochika H (2004). Retrotransposon-induced mutations in grape skin color. Science.

[29] Kofler R, Gómez-Sánchez D, Schlötterer C (2016). PoPoolationTE2: comparative population genomics of transposable elements using Pool-Seq. Mol Biol Evol.

[30] Kofler R, Orozco-terWengel P, De Maio N (2011). PoPoolation: a toolbox for population genetic analysis of next generation sequencing data from pooled individuals. Plos One.

[31] Lerat E, Goubert C, Guirao-Rico S (2019). Population-specific dynamics and selection patterns of transposable element insertions in European natural populations. Mol Ecol.

[32] Linheiro RS, Bergman CM (2012). Whole genome resequencing reveals natural target site preferences of transposable elements in Drosophila melanogaster. Plos One.

[33] Merenciano M, Iacometti C, González J (2019). A unique cluster of roo insertions in the promoter region of a stress response gene in Drosophila melanogaster. Mob DNA.

[34] Miele V, Penel S, Duret L (2011). Ultra-fast sequence clustering from similarity networks with SiLiX. BMC Bioinformatics.

[35] Naito K, Zhang F, Tsukiyama T (2009). Unexpected consequences of a sudden and massive transposon amplification on rice gene expression. Nature.

[36] Nelson MG, Linheiro RS, Bergman CM (2017). McClintock: an integrated pipeline for detecting transposable element insertions in whole-genome shotgun sequencing data. G3.

[37] Platzer A, Nizhynska V, Long Q (2012). TE-locate: a tool to locate and group transposable element occurrences using paired-end next-generation sequencing data. Biology.

[38] Quinlan AR, Hall IM (2010). BEDTools: a flexible suite of utilities for comparing genomic features. Bioinformatics.

[39] Rahman R, Chirn G, Kanodia A (2015). Unique transposon landscapes are pervasive across Drosophila melanogaster genomes. Nucleic Acids Res.

[40] Rice P, Longden I, Bleasby A (2000). EMBOSS: the european molecular biology open software suite. Trends Genet.

[41] Rishishwar L, Mariño-Ramírez L, Jordan IK (2017). Benchmarking computational tools for polymorphic transposable element detection. Brief Bioinform.

[42] Sanseverino W, Hénaff E, Vives C (2015). Transposon insertions, structural variations, and snps contribute to the evolution of the melon genome. Mol Biol Evol.

[43] Slotkin RK, Martienssen R (2007). Transposable elements and the epigenetic regulation of the genome. Nat Rev Genet.

[44] Stewart C, Kural D, Strömberg MP (2011). A comprehensive map of mobile element insertion polymorphisms in humans. PLoS Genet.

[45] Studer A, Zhao Q, Ross-Ibarra J, Doebley J (2011). Identification of a functional transposon insertion in the maize domestication gene tb1. Nat Genet.

[46] Ullastres A, Merenciano M, González J. Natural transposable element insertions drive expression changes in genes underlying Drosophila immune response. BioRxiv. 2019, 655225.

[47] Zhang J, Chen L-L, Sun S (2016). Building two indica rice reference genomes with PacBio long-read and Illumina paired-end sequencing data. Sci Data.

[48] Zhuang J, Wang J, Theurkauf W, Weng Z (2014). TEMP: a computational method for analyzing transposable element polymorphism in populations. Nucleic Acids Res.

