# Supplementary figures and images for: A benchmark of transposon insertion detection tools using real data
Source: Mob DNA. 2019 Dec 30;10:53. doi: 10.1186/s13100-019-0197-9 (PMC6937713; doi:10.1186/s13100-019-0197-9)

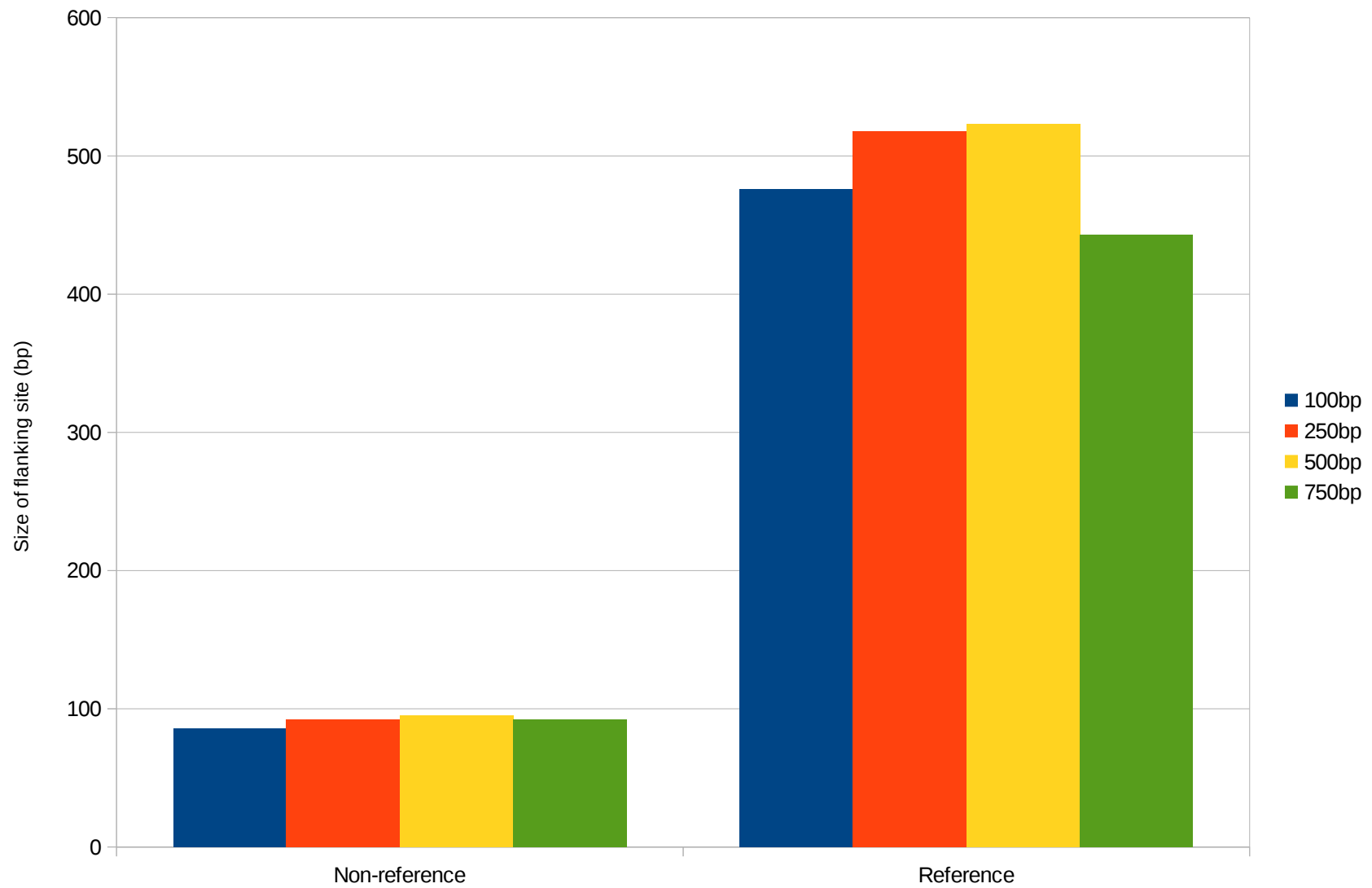

Supplement: Supplementary file 6 — Additional file 6 : Figure S1. Number of MH63 reference and non-reference insertions detected by direct comparison of 1000 LTR-retrotransposon flanking sites of different sizes from MH63 and Nipponbare genomes. (.pdf) [file 13100_2019_197_MOESM6_ESM.pdf]

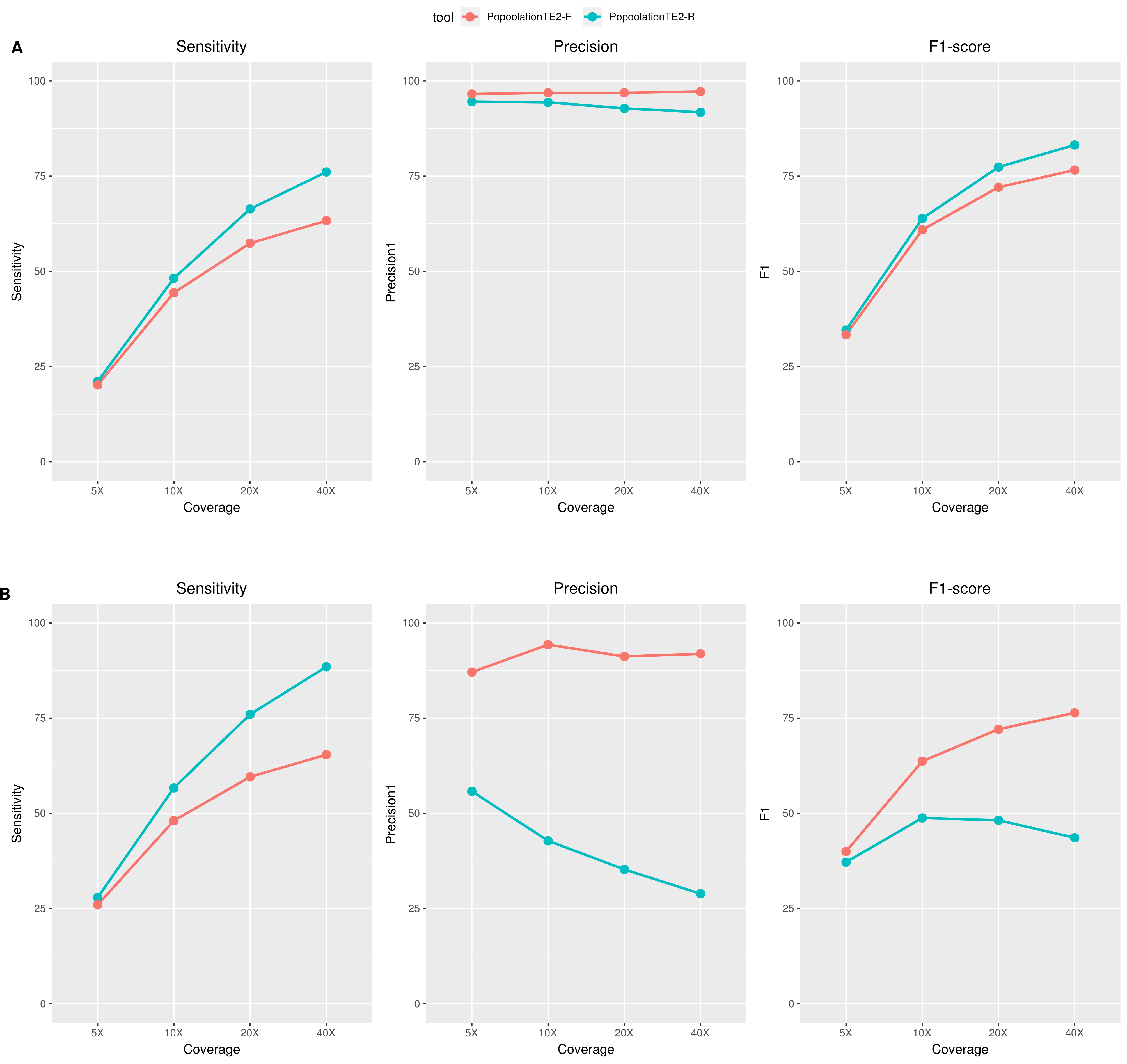

Supplement: Supplementary file 7 — Additional file 7 : Figure S2. Application of zygosity filtering to PoPoolationTE2. PoPoolationTE2-F means that it was run and filtered at zygosity 0.7. PopoolationTE2-R corresponds to the raw results. (.png) [file 13100_2019_197_MOESM7_ESM.png]
